# Anticonstriction Effect of MCA in Rats by Danggui Buxue Decoction

**DOI:** 10.3389/fphar.2021.749915

**Published:** 2021-11-08

**Authors:** Ying Guo, Yating Zhang, Ya Hou, Pengmei Guo, Xiaobo Wang, Sanyin Zhang, Peng Yang

**Affiliations:** ^1^ School of Basic Medical College, Chengdu University of Traditional Chinese Medicine, Chengdu, China; ^2^ Chengdu Fifth People's Hospital, Chengdu, China; ^3^ School of Pharmacy, Chengdu University of Traditional Chinese Medicine, Chengdu, China; ^4^ State Key Laboratory of Southwestern Chinese Medicine Resources, Innovative Institute of Chinese Medicine and Pharmacy, Chengdu University of Traditional Chinese Medicine, Chengdu, China; ^5^ Chengdu Integrated TCM and Western Medicine Hospital, Chengdu, China

**Keywords:** Danggui Buxue decoction, middle cerebral artery, vascular tone, K^+^ channel, Ca^2^^+^ channel

## Abstract

**Objective:** Danggui Buxue decoction (DBD), consisting of Angelicae Sinensis Radix (ASR) and Astragali Radix (AR), is a famous prescription with the function of antivasoconstriction. This study intends to probe its mechanisms on the relaxation of the middle cerebral artery (MCA).

**Methods:** Vascular tension of rat MCA was measured using a DMT620 M system. First, the identical series of concentrations of DBD, ASR, and AR were added into resting KCl and U46619 preconstricted MCA. According to the compatibility ratio, their dilatation effects were further investigated on KCl and U46619 preconstricted vessels. Third, four K^+^ channel blockers were employed to probe the vasodilator mechanism on KCl-contracted MCA. We finally examined the effects of DBD, ASR, and AR on the vascular tone of U46619-contracted MCA in the presence or absence of Ca^2+^.

**Results:** Data suggested that DBD, ASR, and AR can relax on KCl and U46619 precontracted MCA with no effects on resting vessels. The vasodilator effect of ASR was greater than those of DBD and AR on KCl-contracted MCA. For U46619-contracted MCA, ASR showed a stronger vasodilator effect than DBD and AR at low concentrations, but DBD was stronger than ASR at high concentrations. Amazingly, the vasodilator effect of DBD was stronger than that of AR at all concentrations on two vasoconstrictors which evoked MCA. The vasodilator effect of ASR was superior to that of DBD at a compatibility ratio on KCl-contracted MCA at low concentrations, while being inferior to DBD at high concentrations. However, DBD exceeded AR in vasodilating MCA at all concentrations. For U46619-constricted MCA, DBD, ASR, and AR had almost identical vasodilation. The dilation of DBD and AR on KCl-contracted MCA was independent of K^+^ channel blockers. However, ASR may inhibit the K^+^ channel opening partially through synergistic interactions with Gli and BaCl_2_. DBD, ASR, and AR may be responsible for inhibiting [Ca^2+^]_out_, while ASR and AR can also inhibit [Ca^2+^]_in_.

**Conclusion:** DBD can relax MCA with no effects on resting vessels. The mechanism may be related to ASR’s inhibition of K_ATP_ and K_ir_ channels. Meanwhile, the inhibition of [Ca^2+^]_out_ by DBD, ASR, and AR as well as the inhibition of [Ca^2+^]_in_ by ASR and AR may contribute to dilate MCA.

## Introduction

Vascular diseases with a high fatality rate include cerebrovascular disease, cardiovascular disease, hypertension, and atherosclerosis, causing millions of deaths worldwide every year (George et al., 2015; Cainzos-Achirica et al., 2020). According to the “China Health Statistics Yearbook 2020 (National Health Commission, 2019),” the number of deaths of cerebrovascular diseases in China ranked the third following malignant tumors and heart diseases. As an extension of the internal carotid artery, the middle cerebral artery (MCA) tended to being affected by thromboembolism. Among these, evidence had indicated that MCA damage-evoked cerebral infarction events accounted for more than 80% of all cerebral infarctions (Virani et al., 2021). It was reported that brain disease-involved MCA damages constantly caused abnormal changes in vascular tension, especially abnormal contraction of brain vessels (Garg et al., 2021). It thus directly fluctuated the perfusion pressure and the body’s blood supply circulation (Greenstein et al., 2020; Bai et al., 2021). Changes in these factors can lead to the symptoms such as vasospasm, sensory disturbances, and dyskinesia, which in turn result in abnormal changes in vascular tone (Pantoni, 2010; Lee and Lee, 2011; Mehanna and Jankovic, 2013; Krdžić et al., 2015). Also, this vicious circle will eventually be life-threatening.

At present, the main treatment methods for cerebrovascular diseases (such as cerebral hemorrhage, cerebral infarction, and subarachnoid hemorrhage) were surgical treatment and drug treatment (DeBaun et al., 2020; Hernandez Fustes et al., 2020; Sturiale et al., 2020). Of these, there are three main types of conventional drug therapy: 1) antihypertensive and antidiabetic drugs, such as nifedipine, valsartan, and metformin; 2) anticoagulant therapy, such as aspirin and warfarin; 3) symptomatic treatment of cognitive, emotional, and mental disorders, such as memantine hydrochloride, oxiracetam, and clozapine (Jiang et al., 2020; Li T. et al., 2021). However, taking these drugs for a long term was often accompanied by certain liver and kidney damage, gut reaction, perception abnormalities, and perception barriers (Kalantar-Zadeh et al., 2015; Yang et al., 2020).

**FIGURE 1:**
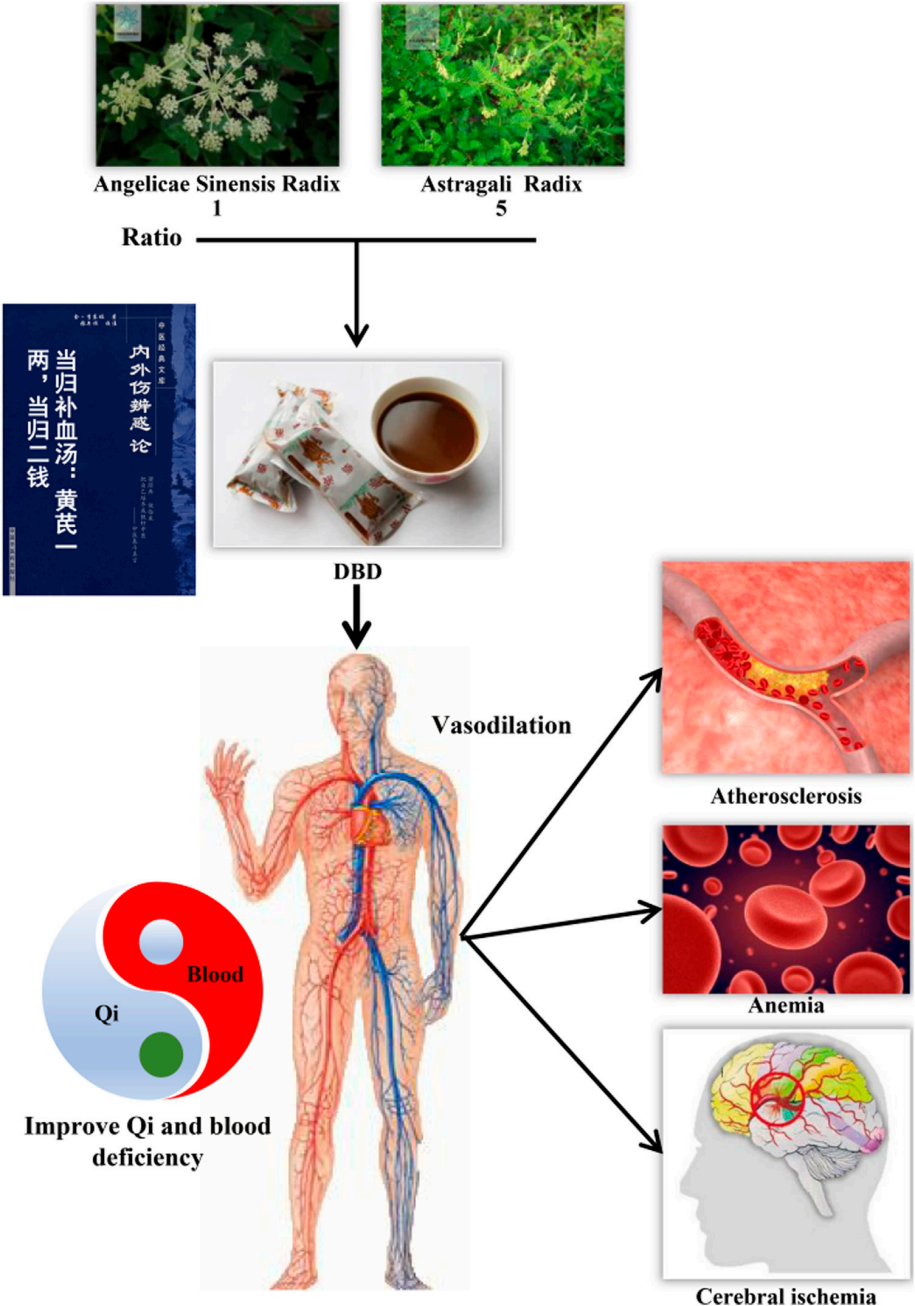
Origin and function of DBD in traditional Chinese medicine. DBD was originally recorded in *Differentiation on Endogenous* (Neiwaishang Bianhuo Lun, 《内外伤辨惑论》), which was composed of AR and ASR in a ratio of 1:5. DBD ameliorated the deficiency of Qi and blood by tonifying Qi and generating blood, thus treating atherosclerosis, anemia, and diseases related to cerebrovascular injuries. AR, Astragali Radix. ASR, Angelicae Sinensis Radix. DBD, Danggui Buxue decoction.

For thousands of years, Chinese people had used traditional Chinese medicine (TCM) to prevent and treat diseases. TCM scholars had found that different herbs may produce better therapeutic effects according to specific combinations. Danggui Buxue decoction (DBD) was originally derived from *Differentiation on Endogenous* (Neiwaishang Bianhuo Lun,《内外伤辨惑论》), which was written by Gao Li in the Song dynasty (Li et al., 2007; Liu et al., 2021). Also, it was composed of the root of Angelica sinensis (Oliv.) Diels (Danggui or Angelicae Sinensis Radix, ASR), and the root of *Astragalus propinquus* and Schischkin (Huangqi or Astragali Radix, AR) are in the ratio of 1:5. In the view of Chinese medicine, it could achieve the purpose of “generating blood” through the way of “replenishing Qi” to treat medical miscellaneous diseases related to Qi deficiency and blood stasis (Lin et al., 2017). Modern pharmacological research found that the classic DBD had the function of hematopoietic characteristics (Yang et al., 2009; Dou et al., 2020; Shi et al., 2020), heart protection (Hu et al., 2018), immunity regulation (Gao et al., 2006a; Gao et al., 2006b), anti-inflammation (Gong et al., 2017; Li C. Y. et al., 2021), and antifibrosis (Chen et al., 2008; Wang and Liang, 2010; Lv et al., 2012). The origin and function of DBD in TCM are shown in Figure 1.

Our research group had found that DBD can promote angiogenesis in rats with myocardial infarction (Hu et al., 2018). In addition, astragaloside IV, a vital component of DBD, could relax the thoracic aorta of rats, and the mechanism involved is related to blocking the Ca^2+^ channel (Hu et al., 2016), which was an important ion channel for the surface of vascular smooth muscles to regulate vascular tension. Experiments have proved that ferulic acid in DBD has the effect of relaxing blood vessels via regulation of the Ca^2+^ channel (Zhou et al., 2017). However, the vasodilator mechanism of DBD remained unclear. Therefore, the purpose of this experiment was to clarify the regulating effect of DBD on vascular tension and to further illustrate whether its vasodilator effect was related to the regulation of K^+^ and Ca^2+^ channels.

## Materials and Methods

### Animals

Healthy male Sprague–Dawley (SD) rats weighing 180–220 g were purchased from Da Shuo Biotechnology Co., Ltd. (Chengdu, Sichuan, China). All rats were housed under identical conditions [the temperature at 25 ± 1°C, the air humidity at 50 ± 5%, and artificial illumination for 12 h (light period 7:00–19:00)]. Commercial solid food and tap water were available *ad libitum* to all animals. All the experimental procedures were performed under the guidelines of the Management Committee from Chengdu University of TCM (Chengdu, Sichuan, China) (Record No. 2015–03).

### Herbs and Reagents

AR and ASR were purchased from the Affiliated Hospital of Chengdu University of TCM and were identified as authentic medicinal materials by Professor Sanyin Zhang of Chengdu University of TCM, and the medicinal materials meet the inclusion requirements of the 2020 Chinese Pharmacopoeia (Chinese Pharmacopoeia Commission et al., 2020). Tetraethtylamine (TEA, no. 134473), 4-aminopyridine (4-AP, no. 275875), BaCl_2_ (no. 342920), glyburide (Gli, no. Y0001511), ethylene glycol-bis (2-aminoethylether)-N, N, N′, N′-tetraacetic acid (EGTA, no. E3889), and 9, 11-dideoxy-9α, 11α-methanoepoxy prostaglandin F2α (U46619, no. D8174) were purchased from Sigma. Astragaloside IV (no. wkq16042601) and ferulic acid (no. wkq17022303) were provided by Weikeqi Biological Technology Co., Ltd. Formononetin (no. DST201025-011) and ligustalide (no. DST200610-007) were procured from Lemeitian medicine.

### Preparation of Lyophilized Powder

The strict quality control in the preparation of lyophilized powder must be carried out to ensure the consistency of drug quality during the research process. First, AR and ASR were pulverized using a pulverizer (RS-FS1401 grinder, Royalstar, China). Therefore, we accurately weighed ASR and AR according to the ratio of 1: 5. The mixture with 10 times the volume of distilled water was boiled at 100°C for 1 h, and then, the supernatant was acquired by centrifugation at 5,000 rpm for 10 min. Afterward, the medicinal material precipitation was added with 5 times the volume of distilled water to repeat the above process. The supernatant was placed in a −80°C refrigerator to freeze overnight. Lyophilized powder of DBD was acquired using a freeze dryer (Eyel4 model freeze dryer, physicochemical Tokyo, Japan). Conformably, ASR and AR freeze-dried powders were prepared by the same method described above. These lyophilized powders were stored in the refrigerator at −20°C. Before the experiment, the concentration of lyophilized powder was prepared in 1 g/ml with distilled water. After centrifugation at 5,000 rpm for 10 min, the supernatant was filtered using a 0.22 μm microporous membrane. According to the minimum and maximum concentrations of DBD in dilating the MCA of rats in our pre-experiment (data were not provided), its series concentrations (8, 16, 32, 64, 128, and 256 mg/ml) were set to the following measures of vascular tension.

### HPLC Analysis

High-performance liquid mass spectrometry (HPLC, Shimadzu L2030) was employed to determine the contents of astragaloside IV, ferulic acid, formononetin, and ligustalide in lyophilized powder of DBD (Jin et al., 2019). First, 0.1% formic acid–water (aqueous phase) and methanol (organic phase) were used as mobile phases, wherein the total flow rate was set at 1 ml/min with a column temperature of 303 K using a C18 column (Agilent 5 HC-C18). The UV detection wavelengths of the above four standards were set at 201 nm (astragaloside IV), 280 nm (ferulic acid and formononetin), and 338 nm (ligustilide). Afterward, all standards (10–20 mg) were diluted to 1 ml, which was defined as mother liquor. Take a certain amount of mother liquor and dilute it by 3.3, 10, 25, 50, and 100 times and filter and perform HPLC tests. All samples were analyzed three times to obtain the standard curves according to the relationship between peak area and concentration. The yield of the component concentration in DBD was determined by comparison with standard calibration curves.

### MCA Vascular Preparation

The PSS liquid (mmol/L: 118 NaCl, 4.7 KCl, 2.5 CaCl_2_, 1.2 KH_2_PO_4_, 1.2 MgCl_2_, 2.5 NaHCO_3_, 11 glucose, and 5 HEPES) was inflated to the saturation state with 95% O_2_ + 5% CO_2_ gas before the experiment. After sacrificing by neck removal, the skull of SD rats was stripped and the brain tissue was removed after bloodletting. The brain tissue was then placed in a Petri dish containing the 4°C cold PSS liquid. After washing the excess blood on the brain tissue with the PSS fluid, the MCAs of rats were then dissected and isolated under a light microscope (XTL-2400 optical microscope, Oka, China). The vessels were cut to 2–3 mm and fixed in the DMT 620 M slot with two 20 μm tungsten filaments, and the temperature was maintained at 37°C. After stabilizing for 20 min, the initial tension of MCA with 1.2 mN was acquired via adjusting the tension button gently four times in a row and being stable for 40 min. Then, the vascular activity was examined by constricting twice with the KPSS liquid (mmol/L: 58 NaCl, 60 KCl, 2.5 CaCl_2_, 1.2 KH_2_PO_4_, 1.2 MgCl_2_, 2.5 NaHCO_3_, 11 glucose, and 5 HEPES), 10 min each time. After each contraction, the vessels were cleaned with the PSS fluid at least three times for 10 min each time to restore the tension as basal tension. The MCA with a vasoconstriction amplitude less than 15% and a vasotension greater than 1 mN between two independent KPSS stimuli was employed for further experiments. The values of vascular tension were recorded by using Labchart Pro professional software v8.3 (ADInstruments, Australia). The effects of all tested drugs at different concentrations on vascular tone were recorded for at least 10 min and repeated five times with different blood vessels.

### Measurement for the Tension of DBD, ASR, and AR on Resting Vessels

The well-activated and eligible MCA vessels were used to investigate the effects of DBD, ASR, and AR at the concentrations of 8, 16, 32, 64, 128, and 256 mg/ml.

### Measurement for the Tension of DBD, ASR, and AR on KCl Preconstricted Vessels

Referring to the previous method (Hu et al., 2016), the vessels of MCA were constricted with 60 mM KCl. Also, the vascular tension of DBD, ASR, and AR (8, 16, 32, 64, 128, and 256 mg/ml) on the KCl preconstricted MCA was recorded. Parallelly, KCl-constricted MCA vascular tension was also measured by the addition of ASR (1.5, 3, 6, 12, 24, 48 mg/ml) and AR (6.5, 13, 26, 52, 104, and 208 mg/ml), consistent with the DBD compatibility ratio.

### Measurement for the Tension of DBD, ASR, and AR on U46619 Preconstricted Vessels

Similarly, another vasoconstrictor U46619 (thromboxane A2 analogue, TXA2; 1 μM) was used to stimulate the MCA contraction (Lv et al., 2014). We further detected the variations in vascular tone by incubation with DBD, ASR, and AR (8, 16, 32, 64, 128, and 256 mg/ml) as well as ASR (1.5, 3, 6, 12, 24, and 48 mg/ml) and AR (6.5, 13, 26, 52, 104, and 208 mg/ml).

### The Effect of K^+^ Channel Blockers on the Relaxation of DBD, ASR, and AR

Four K^+^ channel blockers 4-AP (1 × 10^–3^ mol/L), BaCl_2_ (1 × 10^–4^ mol/L), TEA, and Gli were administered after 60 mM KCl-evoked MCA vasoconstriction. Then, changes in vascular tone were recorded by adding DBD, ASR, and AR (64 mg/ml).

### Measurement for the Tension of DBD, ASR, and AR on Surged Ca^2+^ in Cytoplasm-Evoked Vasoconstriction

**FIGURE 2:**
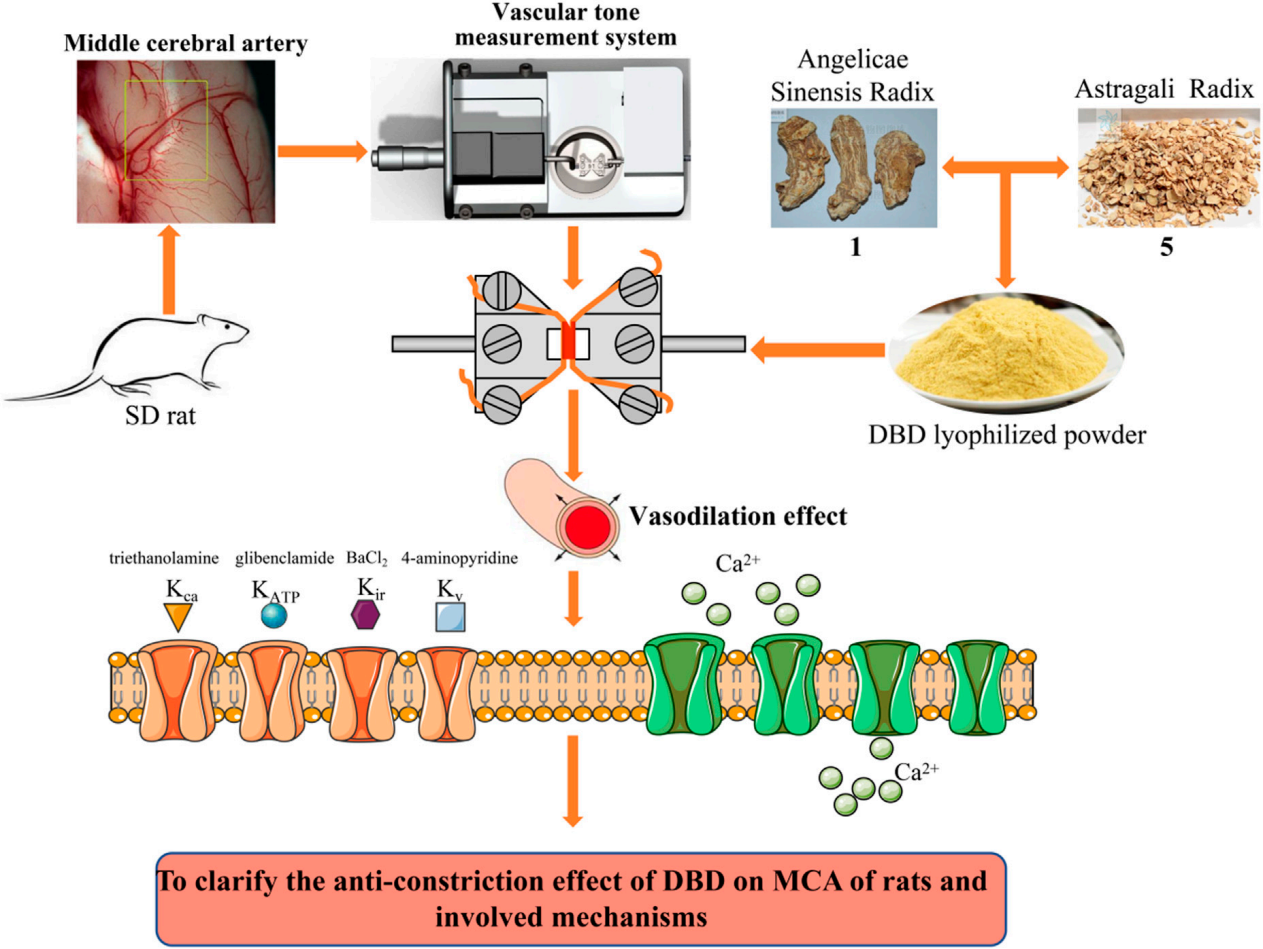
Laboratory technology roadmap. After dissection and isolation of the MCA of rats, the changes of DBD, ASR, and AR on the vascular tension were monitored and recorded using the DMT620 M vascular tension detection system. The potential regulatory activities of K^+^ and Ca^2+^ channels were further demonstrated to clarify the anticonstriction effect by DBD, ASR, and AR. AR, Astragali Radix. ASR, Angelicae Sinensis Radix. DBD, Danggui Buxue decoction. MCA, middle cerebral artery.

According to the previous experimental method (Ma et al., 2020), we evaluated the effects of DBD, ASR, and AR on MCA contraction induced by the release of internal Ca^2+^ from organelles such as the endoplasmic reticulum and mitochondria into the cytoplasmic matrix ([Ca^2+^]_in_). After incubating the MCA vessels with EGTA containing the Ca^2+^-free PSS fluid for 10 min to remove intracellular Ca^2+^, the vessels were then administered with the EGTA- and Ca^2+^-free PSS fluids containing DBD, ASR, and AR (64 mg/ml) for 10 min. Subsequently, 1 μM U46619 was employed to stimulate vasoconstriction and maintained for 10 min. Second, 2.5 mM Ca^2+^ was added to observe whether the above three tested drugs would resist the MCA vasoconstriction induced by the influx of extracellular Ca^2+^ ([Ca^2+^]_out_). The laboratory technology roadmap is shown in Figure 2.

### Statistical Analysis

The experimental data were expressed as the mean ± standard error of the mean (S.E.M). Statistical differences among groups were evaluated by one-way ANOVA with the Tukey–Kramer multiple comparison test using Graph Pad Prism 6.0. *p* < 0.05 was considered statistically significant.

## Results

### HPLC Analysis Results of Four Compounds in DBD

**FIGURE 3:**
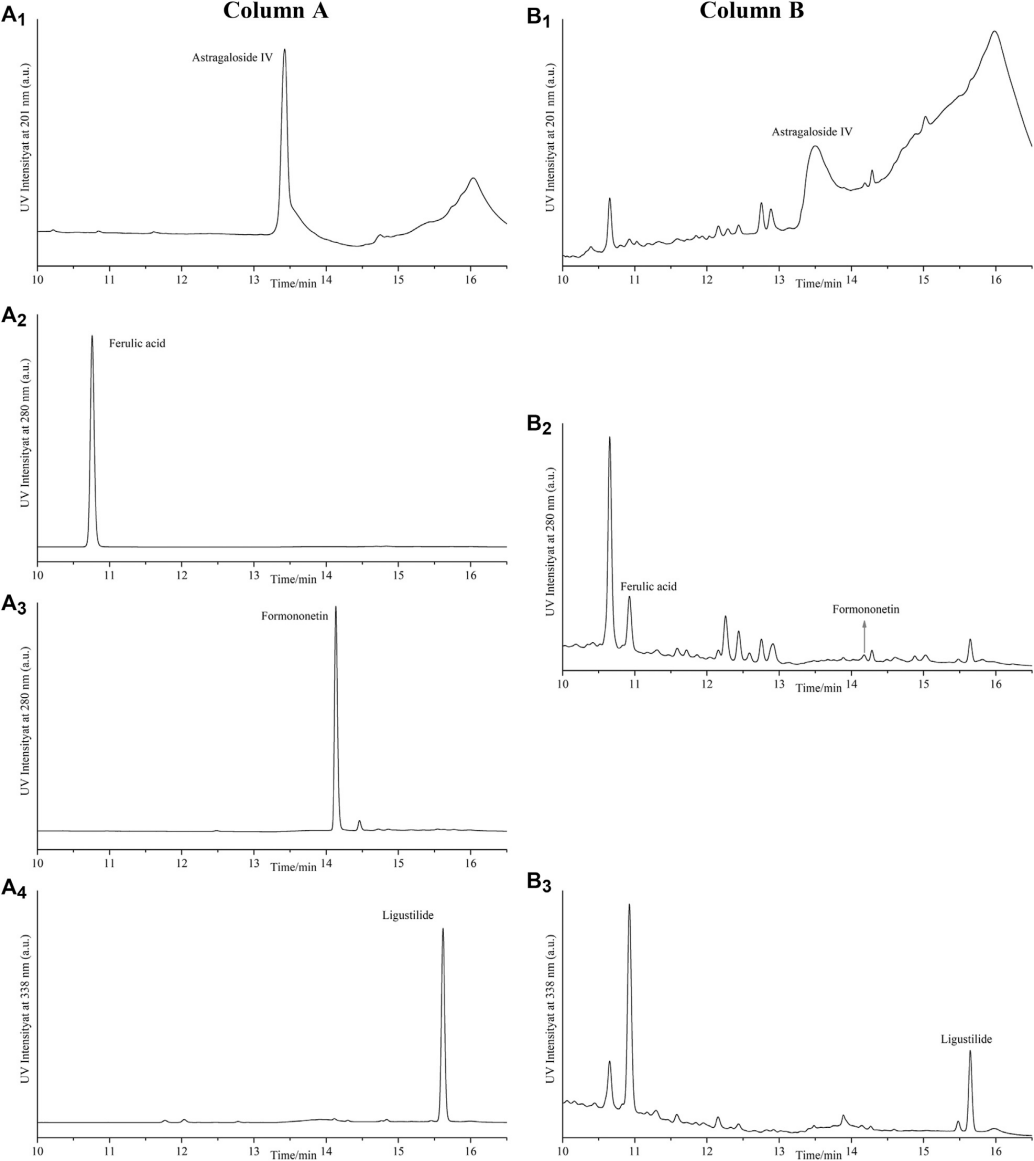
HPLC analysis of four compounds in lyophilized powder of DBD. Column A represents HPLC chromatograms of standard substances astragaloside IV **(A_1_)**, ferulic acid **(A_2_)**, formononetin **(A_3_)**, and ligustalide **(A_4_)**. Column B represents HPLC chromatograms of astragaloside IV **(B_1_)**, ferulic acid, and formononetin **(B_2_)** and ligustalide **(B_3_)** in lyophilized powder of the DBD sample.

Compared with the retention time of the corresponding standards (Figure 3), four compounds in lyophilized powder of DBD were determined by HPLC. As shown in Table 1, the contents of astragaloside IV, ferulic acid, formononetin, and ligustalide were 0.1999, 0.0276, 0.0469, and 1.1296 mg/g, respectively.

**TABLE 1:** Contents of astragaloside IV, ferulic acid, formononetin, and ligustalide in lyophilized powder of DBD identified by HPLC analysis.

NO.	Components	Wavelength (nm)	Retention time (min)	Content (mg/g)
1	Astragaloside IV	201	13.43	0.1999
2	Ferulic acid	280	10.75	0.0276
3	Formononetin	280	14.14	0.0469
4	Ligustalide	338	15.62	1.1296

### DBD, ASR, and AR had No Effects on MCA Under Resting Tension

**FIGURE 4:**
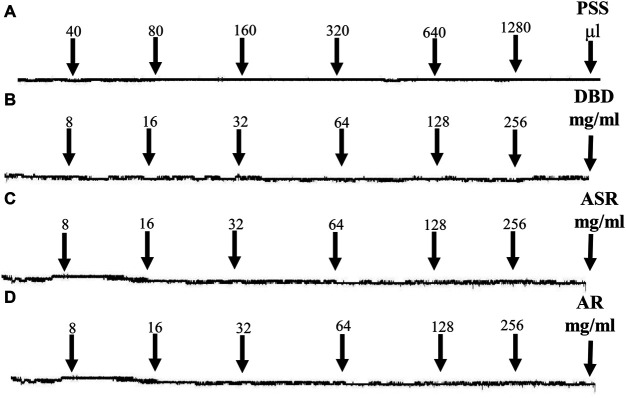
Effects of serial concentrations of DBD, ASR, and AR (8, 16, 32, 64, 128, and 256 mg/ml) on MCA vessels under resting tension. PSS (control, **A**), DBD **(B)**, ASR **(C)**, and AR **(D)** had no effects on resting tension of MCA vessels. DBD, Danggui Buxue decoction. ASR, Angelicae Sinensis Radix. AR, Astragali Radix. MCA, middle cerebral artery.

As shown in Figure 4, PSS solution, as a control, had no effect on MCA vessels under resting tension (Figure 4A). Unanimously, the cumulative addition of DBD (Figure 4B), ASR (Figure 4C), and AR (Figure 4D) (8, 16, 32, 64, 128, and 256 mg/ml) also had no effects on the resting tension of MCA vessels.

### DBD, ASR, and AR Dilated KCl-Evoked MCA Vasoconstriction

**FIGURE 5:**
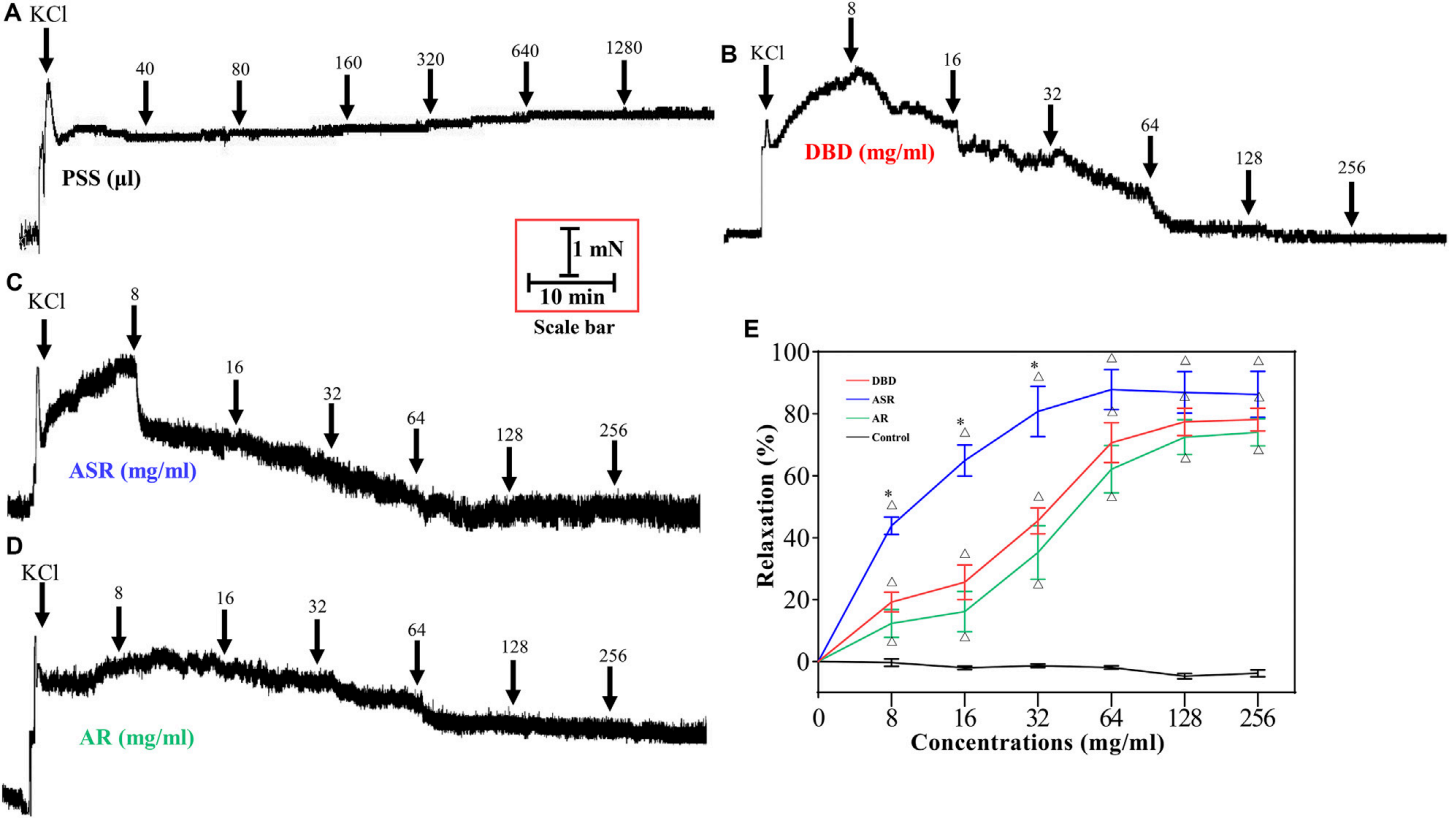
Effect of DBD, ASR, and AR on vascular tension of 60 mM KCl precontracted MCA of rats. Diagrams of the effect of cumulative addition of PSS **(A)**, DBD **(B)**, AR **(C)**, and ASR **(D)** on KCl preconstricted MCA of rats. (E) Comparison of vasodilators by DBD, ARS, and AR on MCA of rats. Data were expressed as the mean ± S.E.M. (n = 5). ^△^ *p* < 0.05 vs the control group; ^*^ *p* < 0.05 vs the DBD group. DBD, Danggui Buxue decoction. ASR, Angelicae Sinensis Radix. AR, Astragali Radix. MCA, middle cerebral artery.

The results in Figure 5 showed the effects of DBD, ASR, and AR on the constricted MCA of KCl. Compared with the control group (Figures 5A and E), DBD (Figures 5B and E), ASR (Figures 5C and E), and AR (Figures 5D and E) (8, 16, 32, 64, and 128 mg/ml) enabled the KCl precontracted MCA to dilate in a concentration-dependent manner (*p* < 0.05). Amazingly, 256 mg/ml of them hardly continued to relax the MCA as compared to the concentration of 128 mg/ml. However, the concentrations of 128 and 256 mg/ml of ASR did not continue to dilate the MCA compared with the concentration of 256 mg/ml (Figures 5C and E).

**FIGURE 6:**
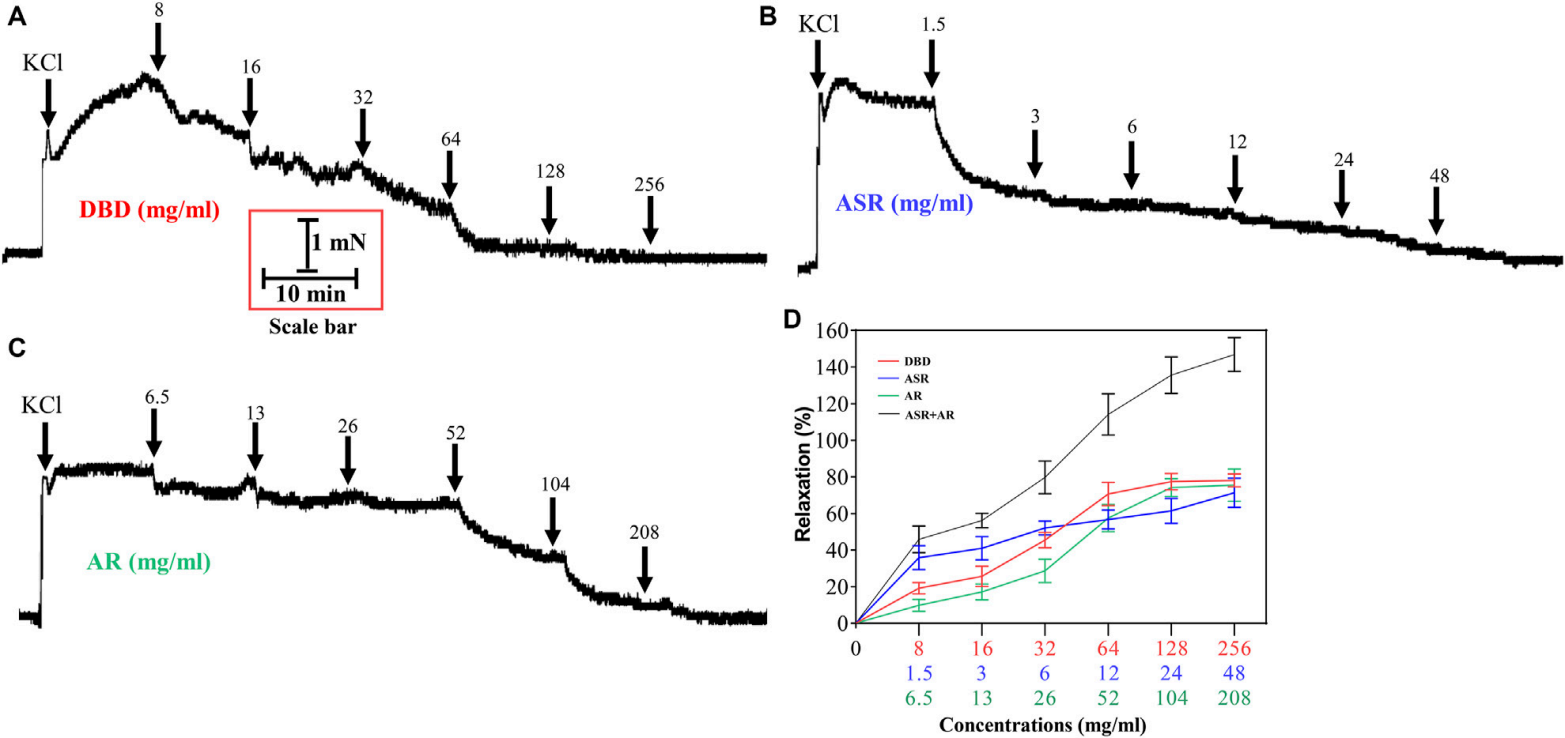
Effect of DBD and its compatibility ratio of ASR and AR monotherapy on the regulation of 60 mM KCl precontracted vascular tension. Diagrams of the effect of cumulative addition of DBD **(A)**, ASR **(B)**, and AR **(C)** on KCl preconstricted MCA of rats. Data were expressed as the mean ± S.E.M. (n = 5). DBD, Danggui Buxue decoction. ASR, Angelicae Sinensis Radix. AR, Astragali Radix. MCA, middle cerebral artery.

To further compare the vasodilator effect of ASR, AR, and DBD on MCA of rats, ASR (1.5, 3, 6, 12, 24, and 48 mg/ml) and AR (6.5, 13, 26, 52, 104, and 208 mg/ml) were added to the MCA vessels constricted by KCl in the prescribed compatibility ratio. Consistently, both ASR and AR can dilate MCA in a concentration-dependent manner, in which the maximum vasodilation rate of ASR at the concentration of 48 mg/ml was 71.28 ± 16.18% (Figures 6B, D), while that of AR at the concentration of 208 mg/ml was 75.52 ± 17.5% (Figures 6B, D). Interestingly, the vasodilator of DBD was not the superimposition of the vasodilator of ASR and AR under the compatibility ratio. At low concentrations (8–16 mg/ml), ASR was superior to DBD in vasodilating MCA, while DBD exceeded AR at concentrations of 128–256 mg/ml.

### DBD, ASR, and AR Dilated U46619-Evoked MCA Vasoconstriction

**FIGURE 7:**
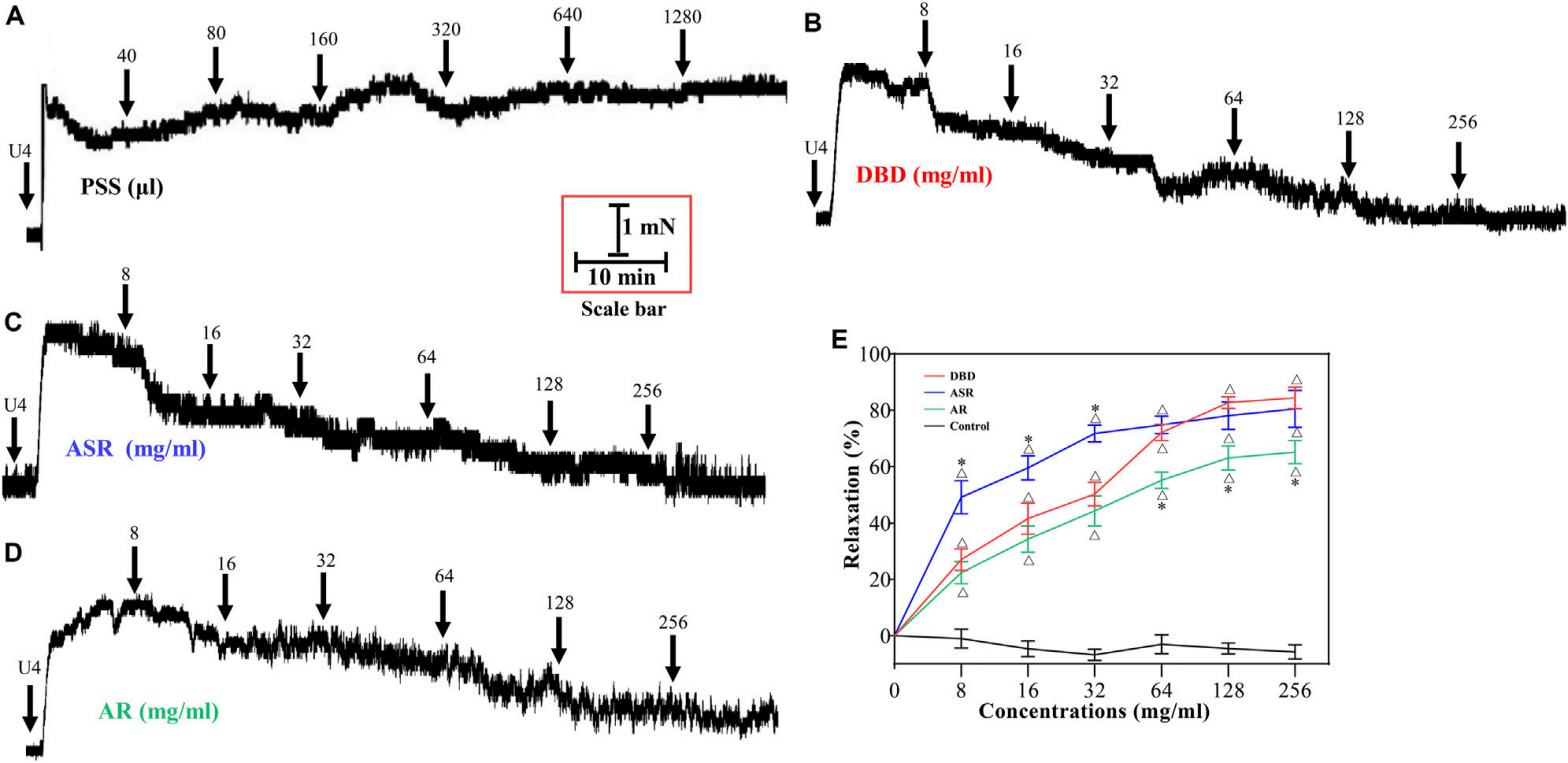
Effect of DBD, ASR, and AR on vascular tension of MCA under 1 μM U46619 precontraction conditions. Diagrams of the effect of cumulative addition of PSS **(A)**, DBD **(B)**, ASR **(C)**, and AR **(D)** on U46619 preconstricted MCA of rats. **(E)** Comparison of vasodilators by DBD, ARS, and AR on MCA of rats. Data were expressed as the mean ± S.E.M. (n = 5). ^△^ *p* < 0.05 vs the control group; ^*^ *p* < 0.05 vs the DBD group. DBD, Danggui Buxue decoction. ASR, Angelicae Sinensis Radix. AR, Astragali Radix. MCA, middle cerebral artery.

**FIGURE 8:**
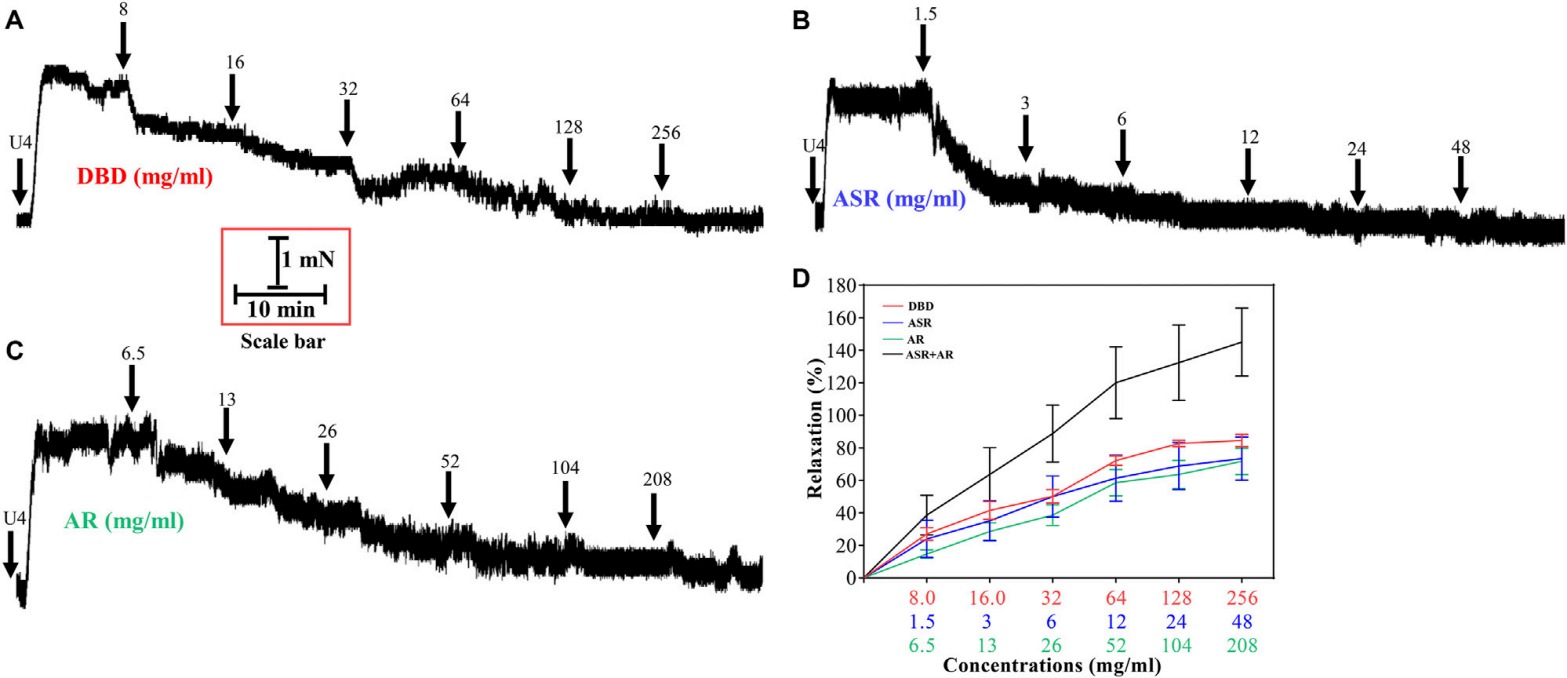
Effect of DBD and its compatibility ratio of ASR and AR monotherapy on vascular tension of MCA under 1 μM U46619 precontraction condition. Diagrams of the effect of cumulative addition of DBD **(A)**, ASR **(B)**, and AR **(C)** on U46619 preconstricted MCA of rats **(D)** Comparison of vasodilators by DBD, ARS, and AR on MCA of rats. Data were expressed as the mean ± S.E.M. (n = 5). DBD, Danggui Buxue decoction. ASR, Angelicae Sinensis Radix. AR, Astragali Radix. MCA, middle cerebral artery.

Similarly, we further investigated the dilatation of DBD, ASR, and AR on U46619-induced MCA contraction in rats. Compared with the control group (Figure 7A), DBD, ASR, and AR all dilated MCA in a concentration-dependent manner with the vasodilator efficiency of ASR > DBD > AR in the concentration range of 8–32 mg/ml and DBD > ASR > AR at the concentrations of 128 and 256 mg/ml (Figures 7B, D). Similarly, ASR and AR disaggregated in prescribed proportions also exhibited concentration-dependent dilation of the constricted MCA vessels of U46619 (Figures 8B, D). However, the vasodilator efficiency of DBD was better than that of ASR and AR within the concentration range we set (Figure 8D) (*p* > 0.05).

### DBD, ASR, and AR Dilated KCl-Constricted MCA Potentially via Inhibiting K^+^ Channel Openness

**FIGURE 9:**
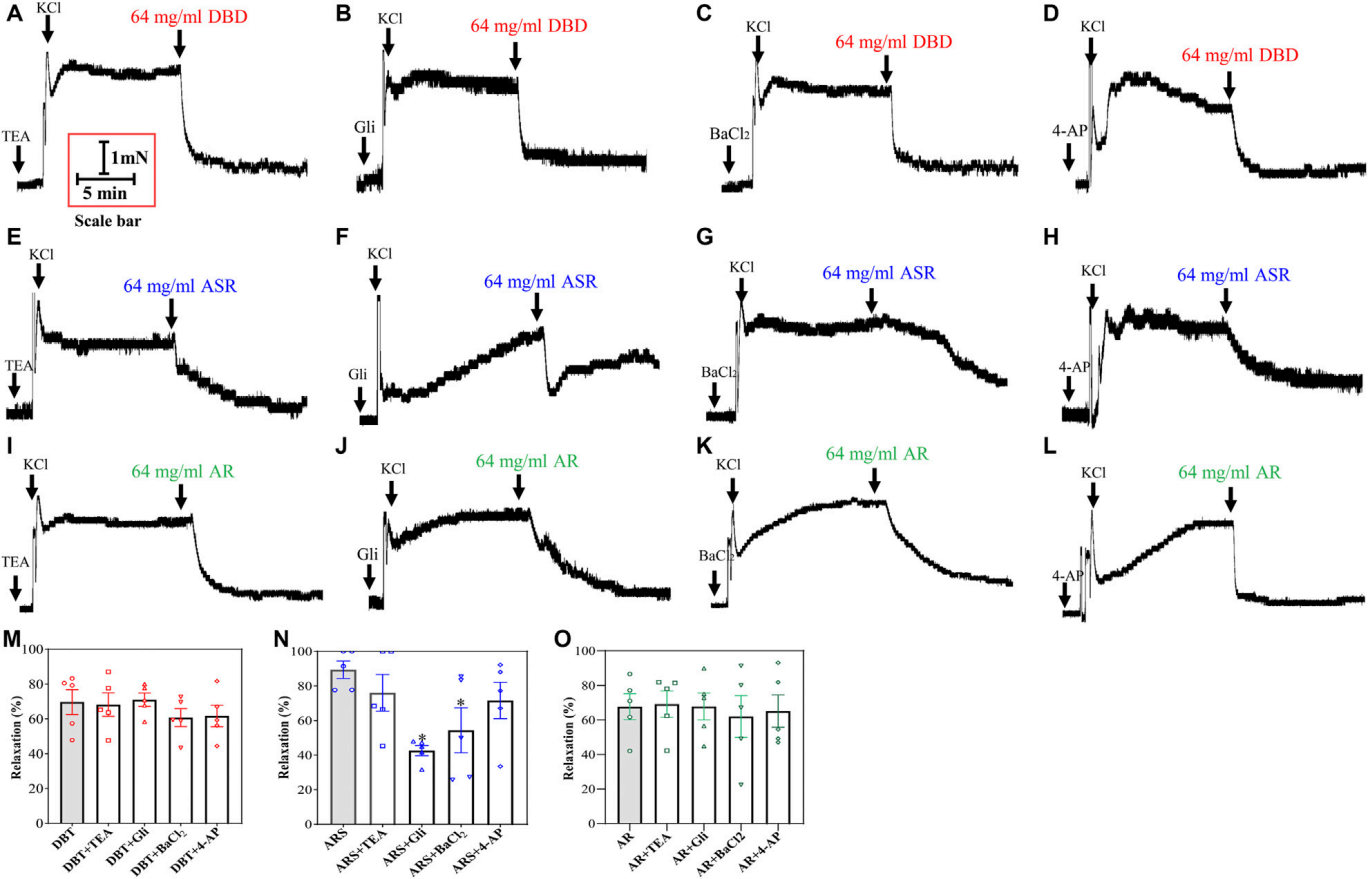
Regulation of DBD, ASR, and AR on K^+^ channels. Potentially inhibiting K^+^ channels opening (TEA, K_Ca_ blocker; Gli, K_ATP_ blocker; BaCl_2_, K_IR_ blocker; 4-AP, K_V_ blocker) by DBD **(A–D**), ASR **(E–H)**, and AR **(I–L)** on 60 mM KCl precontracted MCA of rats. Statistical results and detailed values of DBD **(M)**, ASR DBD **(N)**, and AR DBD **(O)** dilating MCA of rats by regulating four kinds of K^+^ channels. K_Ca_, Ca^2+^-activated K^+^ channel. K_ATP_, ATP-sensitive K^+^ channel. K_IR_, inwardly rectifer K^+^ channel. K_V_, voltage-dependent K^+^ channel. Data were expressed as the mean ± S.E.M. (n = 5). ^*^ *p* < 0.05 vs the control group. DBD, Danggui Buxue decoction. ASR, Angelicae Sinensis Radix. AR, Astragali Radix. MCA, middle cerebral artery.

To probe whether the vasodilator effect of DBD, ASR, and AR is related to the decrease of intracellular K^+^ concentration via the inhibition of K^+^ channel opening, we incubated the KCl-constricted MCA with four K^+^ channel blockers: TEA (1 × 10^–2^ mM, a blocker of Ca^2+^-sensitive K^+^ channels), Gli (1 × 10^–5^ mM, a blocker of ATP-sensitive potassium channels), BaCl_2_ (1 × 10^–4^ mM, a blocker of inwardly rectifer K^+^ channels), and 4-AP (1 × 10^–3^ mM, a blocker of voltage-dependent K^+^ channels). Subsequently, 64 mg/ml of DBD, ASR, and AR were added to investigate their effects on various K^+^ channels. The results showed that the above four kinds of K^+^ channel blockers had no significant effect on the vascular tone of KCl-constricted MCA in rats (Figure 9). In the presence of 4 K^+^ channel blockers, further addition of DBD and AR showed no difference in vascular tone compared with the absence of K^+^ channel blockers (Figures 9A–D, I–M, O) (*p* > 0.05). However, addition of ASR may dilate MCA in a way similar to those of GLI and BaCl_2_ (Figures 9E–H, N) (*p* < 0.05) but not TEA and 4-AP.

### DBD, ASR, and AR Dilated U46619-Constricted MCA Potentially via Inhibiting the [Ca^2+^]_out_ and [Ca^2+^]_in_

**FIGURE 10:**
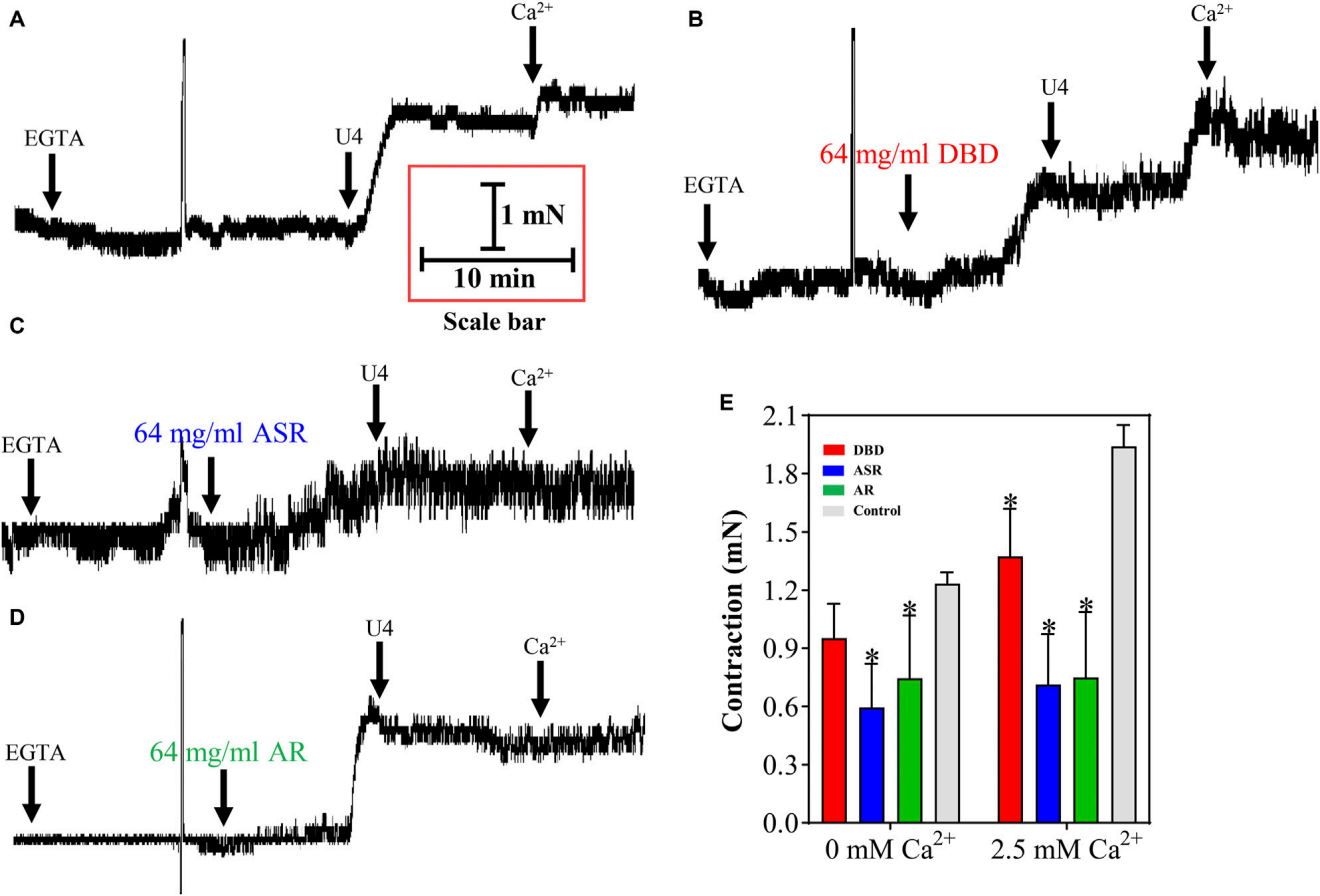
Effect of DBD, ASR, and AR on 1 μM U46619 precontracted MCA of rats by regulation of [Ca^2+^]_out_ and [Ca^2+^]_in_. **(A)** U46619 contraction of vessels without Ca^2+^ and secondary contraction of vessels after recalcification. The effect of DBD **(B)**, ASR **(C)**, and AR **(D)** on vasodilation of MCA in the presence and absence of Ca^2+^. **(E)** Statistical results and detailed values of DBD, ASR, and AR on [Ca^2+^]_out_ and [Ca^2+^]_in_. [Ca^2+^]_out_, the influx of extracellular Ca^2+^. [Ca^2+^]_in_, the release of internal Ca^2+^ from organelles. Data were expressed as the mean ± S.E.M. (n = 5). ^*^ *p* < 0.05 vs the control group. DBD, Danggui Buxue decoction. ASR, Angelicae Sinensis Radix. AR, Astragali Radix. MCA, middle cerebral artery.

We further depleted the Ca^2+^ in the incubation solution to reveal whether the dilation of MCA by DBD, ASR, and AR was related to the inhibition of [Ca^2+^]_out_ or [Ca^2+^]_in_. The results showed that compared with the control group, 1 μM U46619 can induce MCA dilation in the absence of Ca^2+^ in the incubation solution, suggesting that [Ca^2+^]_in_ is involved in this event (Figure 10). Amazingly, preincubation of ASR and AR counteracted U46619-evoked MCA contraction (*p* < 0.05), with the maximum contraction from 1.22 ± 0.07 mN to 0.45 ± 0.39 mN and 0.47 ± 0.38 mN, respectively, indicating the potential inhibition of organelle Ca^2+^ release partly by ASR and AR (Figures 10A, C, D). However, there was no statistically significant difference in DBD in decreasing MCA vascular tone compared with the control group (*p* > 0.05). To demonstrate whether the dilating MCA effect of DBD, ASR, and AR was related to the inhibition of [Ca^2+^]_out_, Figure 10A shows that 2.5 mM CaCl_2_ stimulated the further contraction of MCA. However, the three drugs inhibited the CaCl_2_-mediated secondary contraction of MCA (*p* < 0.05) with the maximum contraction from 1.92 ± 0.13 mN to 1.15 ± 0.22 mN (DBD), 0.52 ± 0.39 mN (ASR), and 0.46 ± 0.4 mN (AR) (Figures 10B–E). The above results collectively suggested that DBD, ASR, and AR may be responsible for inhibiting [Ca^2+^]_out_ against U46619-induced contraction of MCA, while ASR and AR can also inhibit the release of internal Ca^2+^, resulting in the decrease of cytoplasmic Ca^2+^-evoked MCA dilation.

## Discussion

DBD is a TCM prescription used in the treatment of cerebrovascular diseases with a long history. However, there is no evidence to reveal its regulation on the tension of MCA. The results of this experiment revealed the following: 1) vasodilator active ingredients of DBD may be astragaloside IV, ferulic acid, formononetin, and ligustalide. 2) DBD, ASR, and AR have no relaxing effect on the MCA of rats under resting tension. 3) DBD, ASR, and AR can relax the MCA vessels precontracted by KCl and U46619 in a concentration-dependent manner. The vasodilator effect of ASR is characterized by stronger relaxation at low concentrations and rapidly reaches the maximum relaxation rate with the increase of concentration. However, AR has a lower relaxation effect with the relaxation rate slowly increasing. The relaxation effect of DBD is the same as that of AR, but it is stronger than AR at all concentrations. 4) The relaxing effect of DBD on MCA is not the superimposition of ASR and AR. DBD can exert much stronger relaxing effect analogous to ASR at low concentrations as well as the stable relaxing effect analogous to AR on KCl and U46619 precontracted MCA of rats. 5) The vasodilator effect of both DBD and AR may be not related to the K^+^ channel. However, ASR can dilate MCA by the inhibition of K_ir_ and K_ATP_. In addition, ASR and AR can inhibit both the [Ca^2+^]_in_ and [Ca^2+^]_out_, but DBD can only inhibit the [Ca^2+^]_out_.

Cerebrovascular disease was a global disease that caused millions of deaths worldwide every year (Hammond–Haley et al., 2021). In recent years, it has been found that hypertension and vasculitis can both cause cerebrovascular diseases ((Mustanoja et al., 2018; Lersy et al., 2020; and Hou et al., 2021). These diseases are often related to the abnormal contraction of blood vessels, which can cause changes in the body’s homeostasis and further aggravate its abnormal contractions. If this vicious circle is not broken, it will endanger life (Boguslavskyi et al., 2021). Being a very important vessel in the brain, MCA diseases account for a large proportion of all cerebrovascular diseases. Long-term use of traditional drugs for the treatment of cerebrovascular diseases is often accompanied by some adverse reactions. Therefore, the search for drugs that can treat or reduce abnormal vasoconstriction has become a research focus. DBD was used in clinics to treat various medical miscellaneous diseases caused by “deficiency of Qi and blood” (Wu et al., 2016; Chen et al., 2017). Our experiment can thus provide the experimental basis for the better use of DBD.

Concentrations of drugs do result in different pharmacological activities. First, the clinical dosage of DBD was 1 g/kg, and the equivalent dose for rat was 6.3 g/kg. Evidence has shown that astragaloside IV, ferulic acid, formononetin, and ligustilide can be detected in rat plasma after oral administration of DBD (Ji., 2013). Our pre-experimental data confirmed that DBD, lower than 8 mg/ml and higher than 256 mg/ml, had almost no effect on MCA vasodilation in rats with KCl contraction (data were not provided). According to the results of HPLC, the concentrations of astragaloside IV, ferulic acid, formononetin, and ligustilide in 256 mg/ml DBD were 0.0512, 0.0071, 0.012, and 0.2892 mg/ml, respectively. We have previously demonstrated that DBD can promote the proliferation of hypoxic vascular endothelial cells *in vitro* in the range of 3.75–15 mg/ml (Yang et al., 2013). Second, for *in vitro* organs, astragaloside IV (0.01–0.1 mg/ml), ferulic acid (0.1942–0.5825 mg/ml), formononetin (0.0027–0.0268 mg/ml), and ligustilide (0.001–0.4 mg/ml) have been reported to exhibit excellent relaxation on isolated vessels or smooth muscles (Wang et al., 2006; Jia et al., 2014; Li, 2014; Fang et al., 2016; Song, 2018). Our previous investigation indicated that astragaloside IV (0.001–235.491 mg/ml) can dilate MCA of rats (Wang, 2017). At the cellular level, astragaloside IV (0.0075 mg/ml), ferulic acid (0.018 mg/ml), and formononetin (0.0125 mg/ml) showed superior antioxidative stress injury on vascular endothelial cells (Cai et al., 2021). The above evidence in whole-organ cells suggests that the concentrations of DBD (8, 16, 32, 64, 128, and 256 mg/ml) are, to some degree, all reasonable.

Vasoconstrictors are drugs that act on ion channels or specific receptors to cause vascular smooth muscle contraction. KCl-stimulated vasoconstriction is achieved by membrane depolarization (extracellular K^+^ > 20 mM), which activates the opening of voltage-dependent Ca^2+^channels (Hu et al., 2016). TXA2 is mainly produced by prostaglandin H2 in platelets under the action of TXA2 synthase with the effect of promoting platelet aggregation and contraction of vascular smooth muscles (Nguyen et al., 2016). As a representative analogue of TXA2, U46619 can contract vessels via activating cyclic nucleotide-gated channels, causing an increase in intracellular Ca^2+^ and activation of TXA2 receptors (Fang et al., 2016). Therefore, from the molecular level, the mechanisms of the above two vasoconstrictors are completely different. In this experiment, DBD, ASR, and AR did not affect vascular tension without adding vasoconstrictors. This shows that under normal circumstances, the three drugs will not relax uncontracted vessels. The relaxation effects of the three drugs reached the maximum at a certain concentration, indicating that DBD, ASR, and AR all had effects on MCA under the precontraction of the two stimulants. Studies have shown that the important components of DBD, ferulic acid, astragaloside IV, and formononetin can relax coronary arteries (Jia et al., 2014; Fang et al., 2016; and Lin et al., 2018). This is consistent with our experimental results. Dividing the DBD at a certain concentration according to the ratio of ASR: AR = 1:5, it can be found that the relaxing effect of DBD is not the superimposition of the vasodilator effect of ASR and AR. At a lower concentration (8 mg/ml), the relaxation effect of DBD is not stronger than that of the low concentration (1.5 mg/ml) of ASR sinensis at this ratio, but after reaching a certain concentration (64 mg/ml), the relaxing effect of DBD is gradually stronger than that of ASR at this ratio (20.8 mg/ml), which may be caused by the special compatibility mechanism of DBD. Studies have shown that there are differences in the composition of the decoction of different ratios of ASR and AR. The ratio of the classic DBD has been proven to release the effective ingredients better than other ratios (ASR:AR, 1:1, 1:2, 1:3, 1:4, 1:7, 1:10) (Don et al., 2006). This may be the reason why DBD is composed of a large amount of ASR and AR. The role of DBD in protecting blood vessels is closely related to concentration, which is consistent with previous studies (Yang et al., 2013).

K^+^ is a very important ion in the human body. Most of it is stored in cells and a small amount in the extracellular fluid. K^+^ channels are widely present in body tissues and organs and play a role in maintaining cell resting membrane potential, regulating muscle tension and action potential, and participating in cell membrane repolarization (Ykocki et al., 2017). There are four important K^+^ channels distributed on vascular smooth muscles: K_V_, K_ir_, K_Ca_, and K_ATP_. This study showed that preincubation of the four K^+^ channel blockers did not affect the relaxation effects of DBD and AR, while preincubation of the K_ir_ and K_ATP_ channel blockers Gli and BaCl_2_ reduced the relaxation effects of ASR. It is worth noting that in this experiment, we chose a single concentration (64 mg/ml) for the experiment. At this concentration, the three drugs all show good vasodilation effects. It may be that the vasodilator effect of AR at this concentration can compensate for the effect of blockers on ASR, which may be the reason why DBD is not affected by K^+^ channel blockers.

The contraction and relaxation of vascular smooth muscles are affected by not only K^+^ ions but also the increase of [Ca^2+^]_in_, which can stimulate vasoconstriction (Mamo et al., 2014). This experiment shows that in the absence of Ca^2+^, preincubation of DBD, ASR, and AR will not affect vascular tension, but both ASR and AR can inhibit U46619-induced vasoconstriction, while DBD has no obvious effect. On the other hand, the addition of exogenous Ca^2+^ can cause further contraction of vascular smooth muscles. Preincubation of the three drugs can inhibit the secondary contraction induced by exogenous Ca^2+^. DBD, as a prescription for the compatibility of ASR and AR, did not show a more comprehensive inhibitory effect. The reason for this result may be related to the release of ingredients during prescription preparation and freeze-dried powder preparation. Experiments have shown that DBD decoction can promote the release of astragalus components, and the process of making freeze-dried powder may cause certain changes in the components of DBD (Yan et al., 2010; Tan et al., 2021). Simultaneously, we will further apply vascular organoids coupled with microfluidic mass spectrometry chips to define the potential pharmacological components and deep-level molecular mechanisms of DBD on dilating blood vessels (Wang et al., 2019 and, 2020; Trillhaase et al., 2021).

Collectively, our data demonstrated that DBD, ASR, and AR can dilate the rat MCA. In MCA with KCl contraction, the vasodilatation was ASR > DBD > AR. For U46619-contracted MCA, the vasodilatation was ASR > DBD > AR at concentrations less than 128 mg/ml, while DBD > ASR > AR at concentrations greater than 128 mg/ml. Nevertheless, the deep reason why TCM prescription and decomposed prescription show inconsistent pharmacological activities is worth further exploration. In the ischemic stroke model established by middle cerebral artery occlusion, a 14-day administration of Buyang Huanwu decoction can significantly ameliorate the neurological function score of rats. Compared to the model group, groups AR, and the combination of ASR, *Paeonia lactiflora*, *Ligusticum chuanxiong*, *Pheretima aspergillum*, *Carthamus tinctorius*, and *Prunus persica* had a tendency to decrease the neurological function score, separately, with no significant difference (Shu and Pan 2017). Guiqi Congzhi decoction was proved to be superior to groups *Ligusticum chuanxiong* and *Pheretima aspergillum*, *Radix Sophorae Flavescentis*, and *Acorus calamus Linn*, as well as AR and ASR in improving the memory capacity of vascular dementia rats (Ma et al., 2018). For *in vitro* investigation of prescription disassembly, both Xiaobanxia decoction and *Zingiber officinale Roscoe* can counteract the isolated ileum contraction induced by acetylcholine, 5-hydroxytryptamine, and histamine in guinea pigs in a concentration dependent manner, and there was almost no difference in their relaxing effects (Lin, 2018). Evidence also indicated that Siwu decoction was better than any single herbs on oxytocin-induced *in vitro* uterine contractions of mice (Zhu et al., 2011). Meaningfully, the results confirmed that DBD, but not ASR, can significantly resist leukopenia induced by ^60^Coγ -ray radiation in mice (Gu, 2009). In terms of regulating blood vessels, it was revealed that DBD, ASR, and AR can inhibit hepatic angiogenesis in rats with nonalcoholic fatty liver disease by decreasing the activity of nitric oxide synthase, while only DBD and AR can reduce the content of nitric oxide (Guo et al., 2014). The above relevant clues suggested that the pharmacological effects of any TCM prescription, including DBD, should not be identified with the numerical superposition of single herbs. Similarly, we cannot figure out, prescription or single herbs from disassembled prescription, what the strength of the action is. It was thus reasonable for us to believe that the synergistic effect of ASR and AR as well the regulation of other physiological functions may be responsible for the distinct vasodilation of DBD on rat MCA. The elucidation of pharmacological effects of TCM prescriptions involves pharmacokinetics as well as the regulation of multi-components on multi-organ functions (Li C. et al., 2021). Maybe the integrative pharmacology-based investigation is something we should learn from (Xu et al., 2021).

## Data Availability

The raw data supporting the conclusions of this article will be made available by the authors, without undue reservation.

## Abbreviations

4-AP, 4-aminopyridine; AR, Astragali Radix; ASR, Angelicae Sinensis Radix; DBD, Danggui Buxue decoction; EGTA, ethylene glycol-bis (2-aminoethylether)-N, N, N′, N′-tetraacetic acid; Gli, glyburide; MCA, middle cerebral artery; TCM, traditional Chinese medicine; TEA, tetraethtylamine; U46619, 9, 11-dideoxy-9α, 11α-methanoepoxy prostaglandin F2α; [Ca^2+^]_in_, internal Ca^2+^ from organelles; [Ca^2+^]_out_, influx of extracellular Ca^2+^.
